# P-2249. Validation of 2-deoxy 2-[^18^F]fluorocellobiose as a Novel Metabolic PET Radiotracer for Specific Detection and Monitoring of Aspergillus Infections *In Vivo*

**DOI:** 10.1093/ofid/ofae631.2402

**Published:** 2025-01-29

**Authors:** Swati Shah, Jianhao Lai, Morteza Peiravi, Sichen Liu, James Townley, Neysha Martinez-Orengo, Mitchell Turner, Falguni Basuli, Rolf Swenson, Dima A Hammoud

**Affiliations:** National Institutes of Health, Bethesda, Maryland; National Institutes of Health, Bethesda, Maryland; National Institutes of Health, Bethesda, Maryland; NIAID, Bethesda, Maryland; National Institutes of Health, Bethesda, Maryland; National Institutes of Health, Bethesda, Maryland; National Institutes of Health, Bethesda, Maryland; National Institutes of Health, Bethesda, Maryland; National Institutes of Health, Bethesda, Maryland; NIH Clinical Center, Bethesda, MD

## Abstract

**Background:**

A major hurdle to the diagnosis of invasive fungal infections (IFIs), mainly caused by *Aspergillus fumigatus* (Af), remains the lack of timely and definite diagnosis. Several filamentous fungi express β-glucosidases (BGL) which convert cellobiose, a disaccharide, into 2 glucose units. Exploiting this pathway, we developed a novel tracer, 2-deoxy 2-[^18^F]fluorocellobiose (FCB) and showed it can specifically detect Af infections *in vivo*, with minimal background signal and radioactivity accumulation in live Af infection (Fig 1), but not in bacterial infection or sterile inflammation. In this study, we expanded the use of FCB to evaluate treatment response in Af infected mice.

Specific detection of Af infection by FCB-PET imaging

**Figure:**
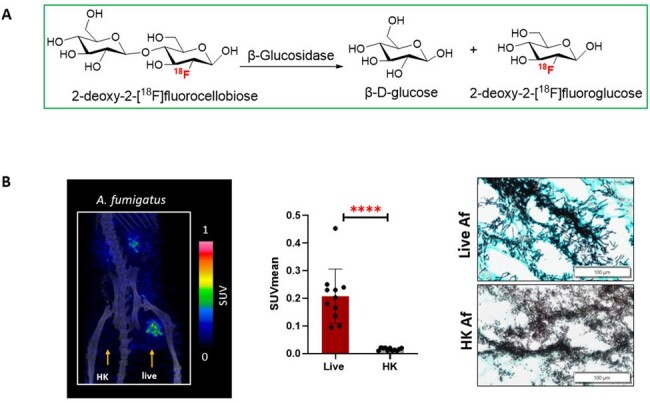

(A) Mechanism of action: FCB in the presence of β-glucosidase (BGL) producing fungi such as A. fumigatus (Af), is metabolized into FDG and glucose molecules. The resulting FDG is retained within the lesions containing metabolically active fungi.

(B) Representative static PET/CT images at 120 mins after ∼7.4-9MBq bolus injection of FCB in mouse myositis models with live (right thigh) or heat-killed (HK) (left thigh) A. fumigatus (n=11) . The signal from the urinary bladder has been removed from the images for clarity purposes. SUVmean values show significantly higher PET signal in the site of live Af infection. ****, p < 0.0001 (unpaired t-test). GMS staining of tissues with live (hyphal structures) and HK (spores) fungi (Accepted for publication).

**Methods:**

FCB was synthesized by enzymatic conversion of 2-deoxy-2-[^18^F]fluoroglucose (FDG). Mice were immunosuppressed using cyclophosphamide. Myositis infections were induced by intramuscular thigh injection of live Af conidia. Daily Voriconazole (VCZ) treatment was started on D1 for 3 weeks (40mg/kg, IP). Longitudinal FCB-PET was performed (D3, D14, D21) and standardized uptake values (SUVmean) were calculated. At the study endpoint, tissues were collected for Grocott’s Methenamine Silver (GMS) staining. Our results were compared to a similar previous treatment study where we used IV Amphotericin B (AmB) (15mg/kg).

Evaluation of treatment response by FCB-PET imaging

**Figure:**
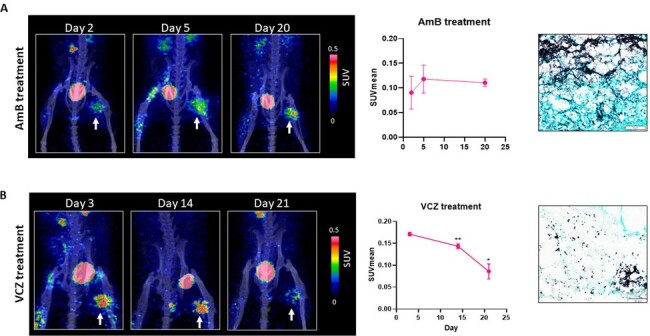

(A) Representative static PET/CT images of FCB uptake and SUV mean values in A. fumigatus myositis models (n=5) at different time-points after amphotericin B (AmB) treatment showing no appreciable decrease in PET signal over time. GMS staining of the thigh muscles at the terminal time-point show proliferating hyphae indicating sub-clinical efficacy of treatment.

(B) Representative static PET/CT images of FCB uptake in A. fumigatus myositis models (n=12) at different time-points after voriconazole (VCZ) treatment showing decreased PET signal over time. SUVmean values show significant decrease in signal corresponding to the live infection focus (ANOVA mixed effects model) with significant treatment effect (p<0.0001). ** p<0.001; *p<0.05. GMS staining shows minimal hyphal growth indicating good control of infection.

**Results:**

FCB-PET showed the initial spread and eventual decline in Af infection in VCZ treated mice with decreasing SUVmean values on D14 and D21 showing significant treatment effect (p< 0.0001, ANOVA mixed effects model) (Fig 2). As expected, VCZ treatment was more effective than AmB treatment which showed sub-clinical efficacy with minimal decrease in FCB-PET signal over time. GMS staining also showed decreased proliferating hyphae in the VCZ cohort unlike the AmB treated group.

**Conclusion:**

We developed a novel Af-specific PET ligand, FCB, and demonstrated its ability to non-invasively diagnose Af infection *in vivo* and monitor treatment effect with different antifungals. Thus, FCB can also be used for repeated efficacy testing of novel therapeutics within the same cohort of animals. FCB is synthesized from commercial FDG and is a highly translatable Af-specific PET ligand. Further usefulness in other fungal infections is being evaluated.

**Disclosures:**

All Authors: No reported disclosures

